# Effect of simultaneous exposure to extremely short pulses of blue and green light on human pupillary constriction

**DOI:** 10.1186/s40101-016-0109-3

**Published:** 2016-08-31

**Authors:** Soomin Lee, Shougo Ishibashi, Yoshihiro Shimomura, Tetsuo Katsuura

**Affiliations:** 1 Center for Environment, Health and Field Sciences, Chiba University, 6-2-1, Kashiwanoha, Kashiwa, 277-0882 Japan; 2 Graduate School of Engineering, Chiba University, Chiba, Japan; 3 Present address: East Japan Railway Company, Tokyo, Japan

**Keywords:** Blue light, LED-pulsed light, Pupillary constriction, Subadditive response

## Background

Light has various influences on all species, including humans. In natural environments, the only light source is sunlight. Humans have been evolving and adapting under such natural light environments. In modern society, illumination in the workplace has a great influence on work efficiency and the health of workers [1]. The effects of illumination are classified as visual effects and non-visual or non-image-forming (NIF) effects. Recently, a number of studies in the field of physiological anthropology have focused on the NIF effects of illumination on humans [2–8].

In 2002, melanopsin-containing intrinsically photosensitive retinal ganglion cells (ipRGCs), a novel type of photoreceptor cells, were found in the mammalian retina [9, 10]. It was confirmed that ipRGCs respond to short-wavelength (blue) light of around 480 nm [9, 11, 12]. The ipRGCs in the retina of the eye affect the interlamellar nuclei of the lateral geniculate nucleus, suprachiasmatic nucleus, intergeniculate leaflet, olivary pretectal nucleus, and ventrolateral preoptic nucleus [10, 13–15] and act as the primary photoreceptors for NIF functions such as melatonin suppression [3, 6, 14, 16–18] and pupillary constriction [5, 7, 8, 14, 18–28].

Recently, it was pointed that the input from cones and rods could potentially affect the ipRGC response [11, 14, 18, 21, 29]. Most vertebrates, including fishes, amphibians, reptiles, and birds, have three or four types of cones and trichromatic or tetrachromatic color vision. However, in the history of evolution, mammals lost a portion of these cones and have dichromatic color vision. Some primates (catarrhines) acquired a third cone and have trichromatic color vision. Humans have three types of cones (S-cones, M-cones, and L-cones) and have trichromatic color vision [30, 31], which is rare in mammals. Figueiro et al. [29] studied the effects of blue (450 nm, 7.7 μW/cm^2^) and green (525 nm, 21.1 μW/cm^2^) light on melatonin suppression at night. They found that simultaneous exposure to blue and green light resulted in less melatonin suppression than monochromatic exposure to blue or green light. This effect is called the subadditive response to light [29]. Figueiro et al. [32, 33] and Revell et al. [34] also identified the subadditive effects of monochromatic and polychromatic light on melatonin suppression, suggesting that cones affected the ipRGC response. However, it remains unclear whether the subadditive response affects pupillary constriction.

The response of mouse ipRGCs to a single photon was examined, and it became clear that ipRGCs have an exceptionally large and prolonged response in comparison with rods and cones [12]. However, ipRGCs are far less sensitive than rods and cones to light intensity [19, 21, 23, 35], so we hypothesized that exposure to high irradiance pulsed light might produce higher NIF function. Therefore, in the present study, we examined the effects of separate and simultaneous exposure to extremely short pulses of blue and green light at different irradiance levels on pupillary constriction and sought to confirm the subadditive response to light.

## Methods

Eleven healthy young Japanese males (mean ± standard deviation age 23 ± 0.9 years, body height 172.7 ± 6.7 cm, body mass 66.2 ± 9.7 kg) with dark eyes participated in this study. They were screened for normal color vision using the Farnsworth-Munsell 100 hue color vision test. Each subject gave his informed consent to participate in the study. The Ethics Committee of the Graduate School of Engineering at Chiba University approved the protocol for the study (#24-25).

**Fig. 1:**
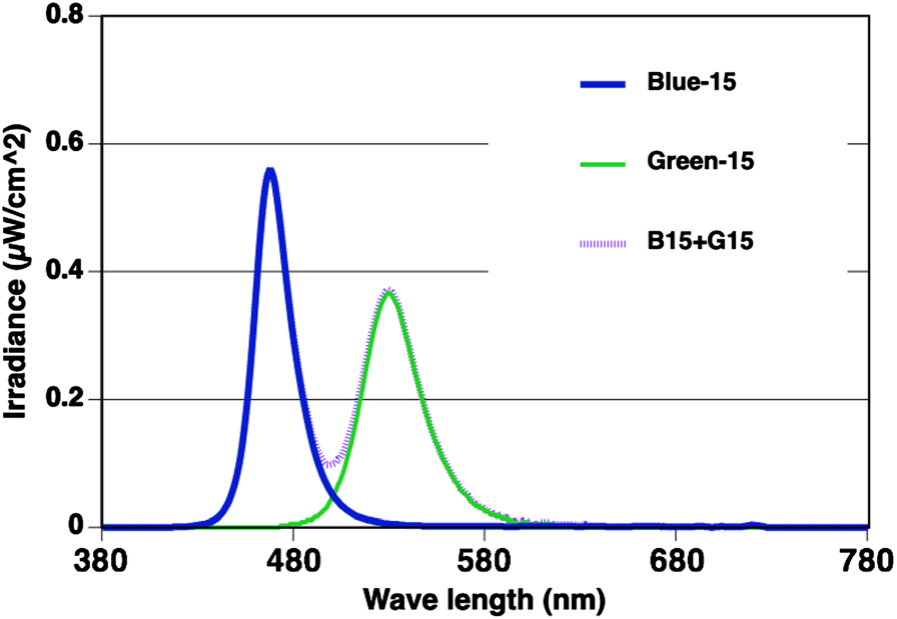
Spectral irradiance of light. Spectral irradiance of blue and green light-emitting diodes and blue plus green light of 15 μW/cm^2^. The light of 10 and 20 μW/cm^2^ irradiance had the same spectral properties

The experiment was conducted in a lighting laboratory controlled at a temperature of 26 ± 0.5 °C and relative humidity of 50 ± 5 %. Each subject sat in a chair with his head facing a diffusion panel, which was located in front of an integrating sphere. Light-emitting diodes (LEDs) were arrayed in the integrating sphere. The spectral irradiance of blue and green LEDs was measured at each subject's eye level using a spectroradiometer (HSR-8100, MAKI Manufacturing, Co. Ltd., Hamamatsu, Japan). The peak wavelength of blue light was 470 nm and that of green light was 532 nm (Fig. 1). Each subject was exposed to nine different light conditions, i.e., a pulse of blue and/or green light of 10, 15, and 20 μW/cm^2^, simultaneously or separately (Table 1). The melanopsin-stimulating irradiance and photon density at the subject’s retinal level were estimated for each light condition [35] based on the spectral absorption of the crystalline lens [36] and a template [37] indicating the spectral absorption characteristics of photopigment with a peak wavelength of 484 nm [9].

**Table 1:** Characteristics of the light conditions

Light condition	Irradiance (*μ*W/cm^2^)	Photon density (10^13^ photons/cm^2^/s)	Photon density (log photons/cm^2^/s)	Melanopsin-stimulating photon density (10^13^ photons/cm^2^/s)
Blue10 (B10)	10.2	2.4	13.4	2.2
Green10 (G10)	10.0	2.7	13.4	1.1
B10 + G10^a^	20.2	5.6	13.7	3.3
Blue15 (B15)	15.4	3.7	13.6	1.7
Green15 (G15)	14.9	4.0	13.6	1.7
B15 + G15^a^	30.3	7.7	13.9	1.7
Blue20 (B20)	19.8	4.7	13.7	4.2
Green20 (G20)	19.9	5.3	13.7	2.3
B20 + G20^a^	39.7	10.0	14.0	6.5

B + G^a^ indicates simultaneous exposure to blue and green light (double irradiance intensity as compared with separate exposure to blue or green light)

**Fig. 2:**
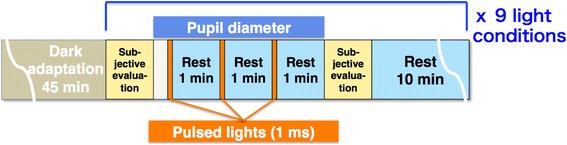
Experimental procedure

After 45 min of dark adaptation (<0.5 lx), the subject was exposed to three pulses of lights with a 1-ms-pulse width in a square waveform every 1 min in each of the nine light conditions. Each subject took a 10-min rest between exposure to each light condition (Fig. 2). The experiments were carried out at 9 a.m. to midday or at 1 p.m. to 4 p.m.. The order of the nine light conditions was counterbalanced among the subjects.

**Fig. 3:**
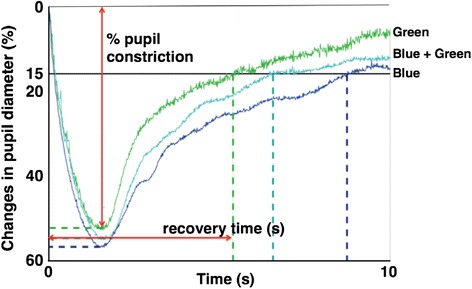
Changes in pupil diameter during separate and simultaneous exposures to pulses of blue and green light. Percent pupil constriction and recovery time were measured

We measured the pupil diameter (EMR-8B, NAC Image Technology Inc., Tokyo, Japan) in the subject’s left eye and used the Kwansei Gakuin Sleepiness Scale (KSS) and the visual analog scale (VAS) for subjective evaluation of “sleepiness.” Pupil diameter measurements during 10 s before and 10 s after exposure to three pulses of light were averaged for each subject and under each light condition. The mean value for the averaged pupil diameter in the 10 s before exposure to the pulses of light was defined as the baseline value. From the averaged pupil diameter (PD) measurement, we calculated the percent pupil constriction and recovery time (Fig. 3) as follows:

$$ \mathrm{Percent}\ \mathrm{pupil}\ \mathrm{constriction} = \left[\left(\mathrm{baseline}\ \mathrm{P}\mathrm{D}\ \hbox{--}\ \mathrm{minimum}\ \mathrm{P}\mathrm{D}\ \mathrm{after}\ \mathrm{exposure}\ \mathrm{t}\mathrm{o}\ \mathrm{pulsed}\ \mathrm{light}\right)\ /\ \mathrm{baseline}\ \mathrm{P}\mathrm{D}\right] \times 100 $$

Recovery time: time (s) until recovery to 15 % pupil constriction after exposure to pulsed light.

KSS and VAS for “sleepiness” were evaluated by the change from the value before exposure to each pulse of light to the value after exposure.

Two-way repeated measures analysis of variance (ANOVA) was applied to clarify the effects of irradiance intensity (10, 15, and 20 μW/cm^2^) and wavelength (blue, green, and simultaneous blue and green). In the case of a significant interaction, one-way repeated measures ANOVA was applied to evaluate the effects of wavelength under each irradiance condition on these measurements. When any significant main effect was found, multiple comparisons of the light condition were performed using the Bonferroni procedure. The data were analyzed using SPSS version 23.0 software (IBM Corp., Armonk, NY, USA). The level of statistical significance was set at 0.05.

## Results

### Percent pupil constriction

Pupillary constriction was observed during exposure to pulsed light under all light conditions. Table 2 shows the results for percent pupil constriction during the nine light conditions. Two-way repeated measures ANOVA revealed that the main effects of irradiance and wavelength on percent pupil constriction were significant (*F*(2, 20) = 14.78, *p* = 0.000 and *F*(2, 20) = 10.79, *p* = 0.001, respectively). However, the interaction effect of irradiance and wavelength on percent pupil constriction was not significant (*F*(4, 40) = 2.19, *p* = 0.087).

**Table 2:** Percent pupil constriction during the nine light conditions

Condition	Blue			Green			Blue + green		
	B10	B15	B20	G10	G15	G20	B10 + G10	B15 + G15	B20 + G20
Mean	41.2	48.6	51.8	32.6	40.3	42.8	43.1	43.3	44.7
SD	8.22	9.43	8.64	9.92	13.37	9.93	8.67	8.18	8.41

Percent pupil constriction was calculated by the following equation: [(baseline PD − minimum PD after exposure to pulsed light) / baseline PD] × 100

*SD* standard deviation

**Fig. 4:**
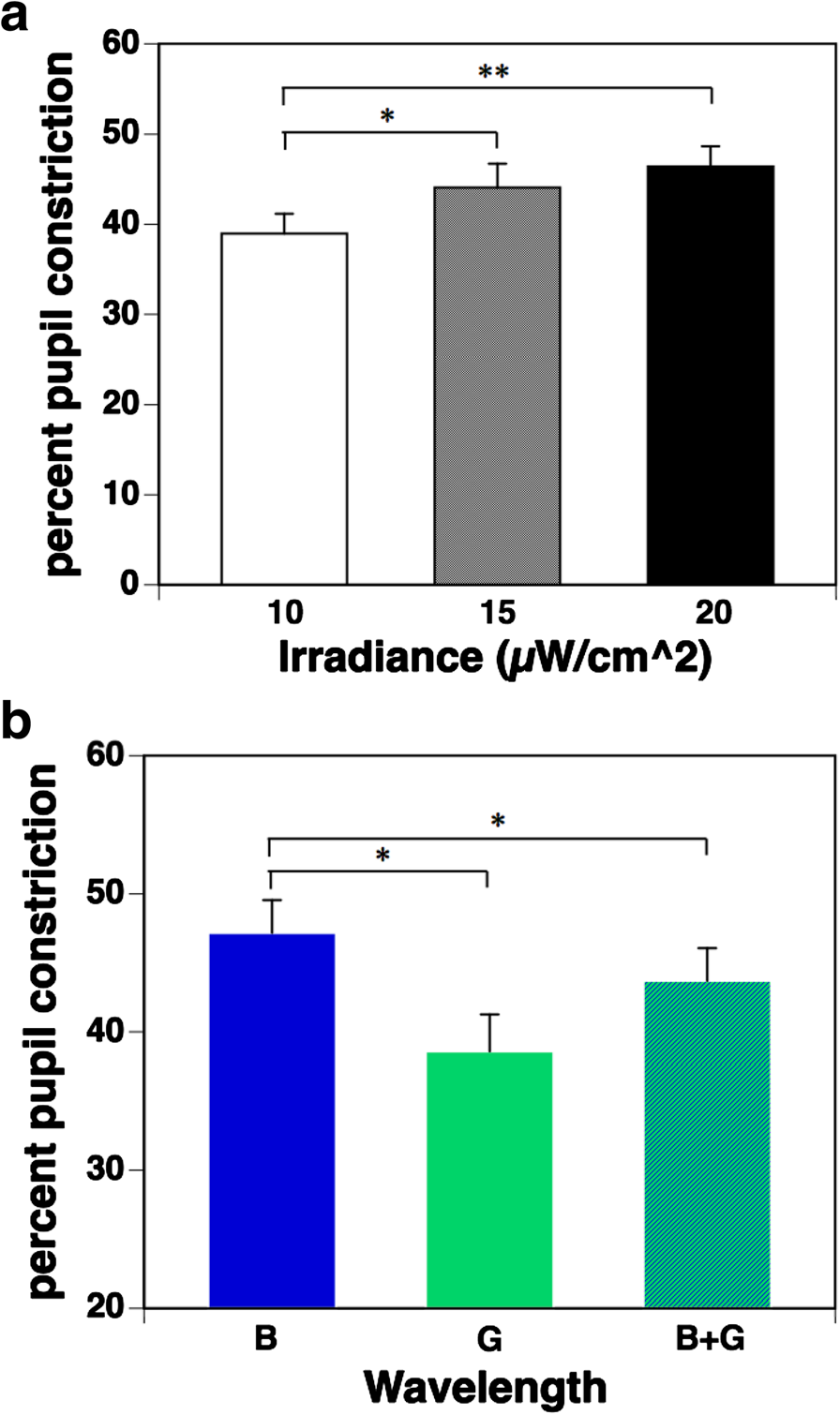
**a** Percent pupil constriction under three irradiance conditions (mean + standard error of the mean). Percent pupil constriction was calculated by the equation [(baseline PD − minimum PD after exposure to pulsed light) / baseline PD] × 100. The main effect of irradiance on percent pupil constriction was significant (*p* < 0.01). Multiple comparisons by the Bonferroni procedure found that irradiance conditions under 15 and 20 μW/cm^2^ resulted in significantly pronounced pupillary constriction when compared with conditions under 10 μW/cm^2^; however, there was no significant difference in those under 15 and 20 μW/cm^2^ irradiance conditions. **b** Percent pupil constriction under three wavelength conditions (mean + standard error of the mean). The main effect of wavelength on percent pupil constriction was significant (*p* < 0.01). Multiple comparisons found that the percent pupil constriction during exposure to a pulse of blue light (B) was significantly more pronounced than during exposure to a pulse of green light (G). Interestingly, the percent pupil constriction during simultaneous exposure to blue and green (B + G) light was significantly inhibited than during exposure to a pulse of blue light (B). *p < 0.05 **p<0.01

Figure 4a shows the percent pupil constriction under three irradiance conditions. Multiple comparisons using the Bonferroni procedure found that 15 and 20 μW/cm^2^ irradiance conditions resulted in significantly more pronounced pupillary constriction than the 10 μW/cm^2^ condition (*p* = 0.014 and 0.005, respectively). However, there was no significant difference between 15 and 20 μW/cm^2^ irradiance conditions (*p* = 0.106).

The percent pupil constriction under the three wavelength conditions is shown in Fig. 4b. Multiple comparisons found that the percent pupil constriction during exposure to a pulse of blue light (B) was significantly (*p* = 0.010) more pronounced than during exposure to a pulse of green light (G). Interestingly, the percent pupil constriction during simultaneous exposure to blue and green (B + G) light was significantly (*p* = 0.031) more inhibited than during exposure to a pulse of blue light (B), despite the double irradiance intensity and the 1.5× melanopsin-stimulating photon density, as shown in Table 1.

### Recovery time

**Fig. 5:**
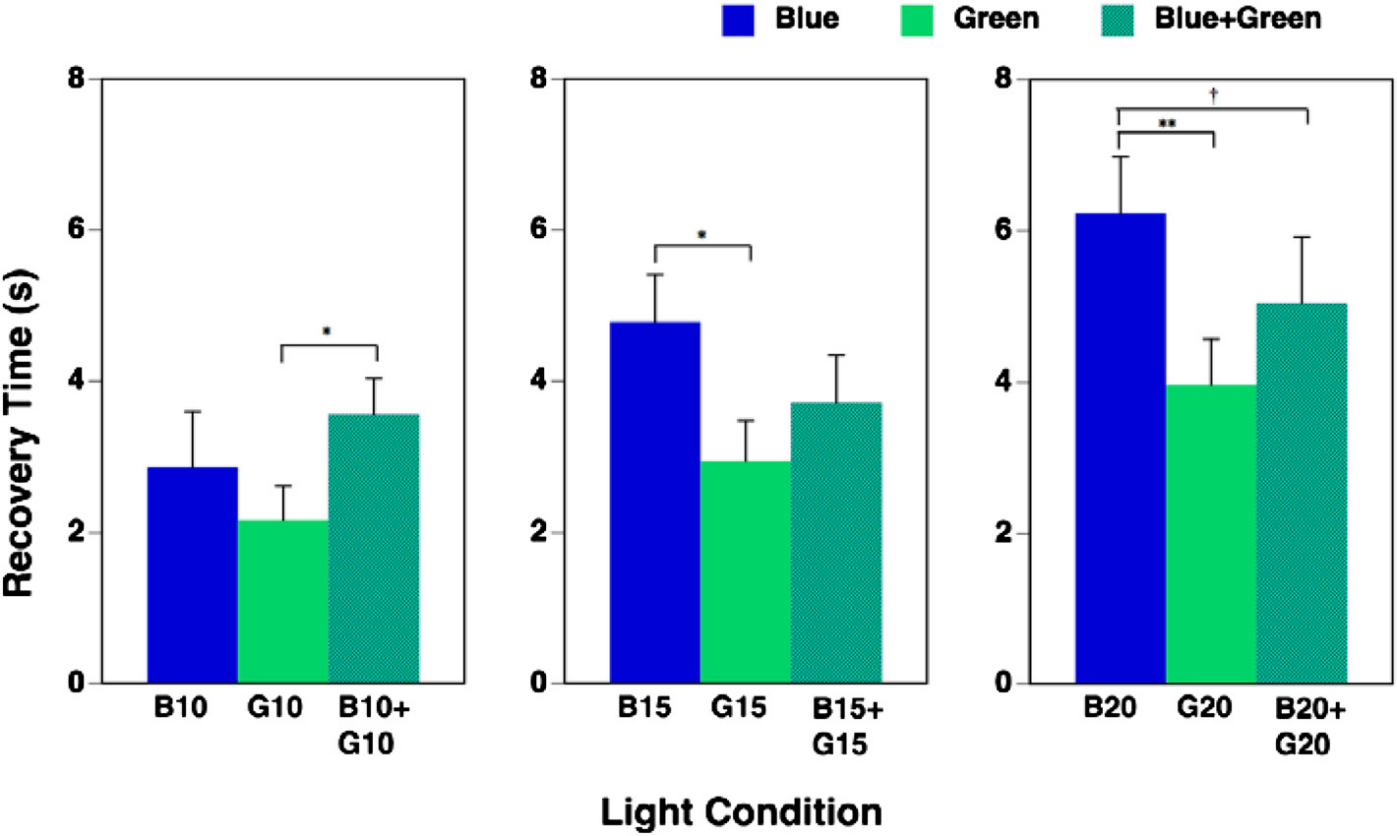
Recovery time during each of nine light conditions (mean + standard error of mean). Recovery time denotes the time (s) until recovery to 15 % pupil constriction after exposure to pulsed light. The main effects of irradiance and wavelength on recovery time was significant (*p* < 0.01), and the interaction of irradiance and wavelength was also significant (*p* < 0.05); therefore, one-way repeated measures ANOVA was applied. The main effects of wavelength on recovery time under each 10, 15, and 20 μW/cm^2^irradiance condition were all significant (*p* < 0.05, *p* < 0.05, and *p* < 0.01, respectively). The multiple comparisons clarified that the recovery of pupillary constriction during exposure to the highest irradiance of simultaneous exposure to blue and green (B20 + G20) light had a tendency (*p* = 0.073) to be longer than during blue light (B20). ^†^ *p* < 0.1, *p < 0.05, **p < 0.01

The results for time taken for pupillary constriction to recover upon exposure to the nine light conditions are shown in Table 3. Two-way repeated measures ANOVA determined that the main effect of irradiance on time taken to recovery was significant (F(2, 20) = 19.08, *p* = 0.000). Higher irradiance resulted in a longer recovery time. The main effect of wavelength was also significant (F(2, 20) = 8.42, *p* = 0.002). The interaction of irradiance and wavelength was significant (F(4, 40) = 3.59, *p* = 0.014), so one-way repeated measures ANOVA was applied to evaluate the effects of wavelength under each irradiance condition on the recovery time. We found that the main effects of wavelength on recovery time under each 10, 15, and 20 μW/cm^2^ irradiance condition were all significant (10 μW/cm^2^: *F*(2, 20) = 3.81, *p* = 0.040; 15 μW/cm^2^: F(2, 20) = 5.46, *p* = 0.013; 20 μW/cm^2^: F(2, 20) = 10.72, *p* = 0.001, respectively). Multiple comparisons showed that recovery of pupillary constriction upon exposure to the highest irradiance of simultaneous exposure to blue and green (B20 + G20) had a tendency (*p* = 0.073) to be longer on exposure to blue light (B20) despite the double irradiance intensity of the combination, as shown in Fig. 5.

**Table 3:** Recovery time of pupillary constriction during the nine light conditions

Condition	Blue			Green			Blue + green		
	B10	B15	B20	G10	G15	G20	B10 + G10	B15 + G15	B20 + G20
Mean	2.94	4.79	6.24	2.17	2.95	3.97	3.57	3.72	5.04
SD	1.66	2.06	2.47	1.47	1.77	2.01	1.56	2.09	2.90

Recovery time denotes the time (s) until recovery to 15 % pupil constriction after exposure to pulsed light

*SD* standard deviation

There was no significant main effect of light condition on subjective evaluations using KSS and VAS scores for “sleepiness.”

## Discussion

We found pupillary constriction under all light conditions used in the present study. It has been suggested that pupillary constriction is controlled mainly by rods under lower irradiance light exposure and by ipRGCs under higher irradiance light exposure [14, 21, 23, 35]. The ipRGCs have been reported to contribute to the pupillary response to light in mice at an irradiance level greater than about 13 log photons/cm^2^/s of 470 nm light at the eye level [23]. It was also reported that the threshold retinal irradiance for depolarization of ipRGCs in rats was about 12.7 log photons/cm^2^/s of 500 nm light [9]. In the present study, the lowest irradiance intensity of blue (B10) was 13.4 log photons/cm^2^/s, which was higher than the threshold intensity for the activation of ipRGC. The lowest irradiance intensity of green (G10) was 13.4 log photons/cm^2^/s; however, the melanopsin-stimulating photon density of G10 was 1.1 photons/cm^2^/s, which was half the value of B10. From the standpoint of the melanopsin-stimulating effect, G10 corresponded to about 5 μW/cm^2^ or 13.1 log photons/cm^2^/s of blue light. This value was still higher than the threshold intensity of ipRGC activation.

In the present study, we used extremely short pulses of monochromatic light with a pulse width of 1 ms. There has been very little research on NIF function using such short pulses of light. We previously conducted an experiment with pulsed blue light (irradiance 11.2 μW/cm^2^, pulse width 100 μs) and continuous blue light (irradiance 1.4 μW/cm^2^), which had the same multiplication value for irradiance and duration, and found that pupillary constriction was significantly greater under the extremely short pulsed light condition than under the continuous light condition [5]. Recently, Vartanian et al. [25] studied pupillary constriction using flickering light stimuli under combined conditions with seven flicker frequencies (0.1, 0.25, 0.5, 1, 2, 4, and 7 Hz), three total photons (13.7, 14.7, and 15.7 log photons/cm^2^), and three duty cycles (12, 47, and 93 %). They found the greatest pupillary constriction was evoked by the stimuli of flickering at 2 Hz with a 12 % duty cycle and 13.7 log photons/cm^2^ conditions, which was 71 % greater than that evoked by equal-intensity (12.3 log photons/cm^2^/s) continuous light. This frequency and duty cycle were also optimal for 14.7 log photons/cm^2^ stimuli, which was 38 % greater than that evoked by equal-intensity constant light. The pulse width of the stimuli at 2 Hz with a 12 % duty cycle is 60 ms. Although the pulse width in the study by Vartanian et al. [25] was much longer than that used in the present study, their results suggest that pulsed light has a greater influence on pupillary constriction.

We also found that pupillary constriction during exposure to pulsed blue light was significantly greater than during exposure to pulsed green light. These results are in accordance with those of a previous study [27] comparing pupillary constriction during 45 s of exposure to continuous blue and green monochromatic light at three irradiance intensities after 15 min of dark adaptation. The greater pupillary constriction during exposure to blue light might be involved in the higher melanopsin-stimulating photon density of blue light.

In the present study, the most important finding was that pupillary constriction during simultaneous exposure to blue and green light was significantly decreased when compared with separate exposure to blue light, despite the double irradiance intensity and a 1.5× melanopsin-stimulating photon density. Recovery of pupillary constriction during 20 μW/cm^2^ of simultaneous exposure to blue and green light (B20 + G20) also had a tendency to be more rapid than during separate exposure to blue light (B20). These findings indicate that the effect of blue light on ipRGCs is inhibited by simultaneous exposure to green light.

It has been reported that ipRGCs receive synaptic input from rods and cones [11, 14, 18, 21, 29]. Information on light wavelength relayed by the rods and the three types of cones is processed by bipolar cells, horizontal cells, and amacrine cells in the retina [38]. It is well known that the human visual system segregates cone responses into color information processed by two channels [32], one class of bipolar cells forms the red versus green (r/g) channel with opposing input from L-cones and M-cones, and the other class forms the blue versus yellow (b/y) channel from S-cones opposed to the combined input from the L-cones and M-cones and brightness information in the ganglion cells; and this information is sent to the visual cortex via the optic nerve [29]. In humans, spectral opponent blue versus yellow (b/y) bipolar cells have been hypothesized to provide direct input to the ipRGCs [29]. In fact, the ipRGCs in the in vitro primate retina show an unusual “color-opponent” receptive field in which an S-cone-mediated OFF response is antagonistic to an (L + M)-cone-mediated ON response on electrophysiological recordings [11], and an S-cone-mediated ON response is opposed to an (L + M)-cone-mediated OFF response in a similar way [39]. Thus, a b/y pathway originates in the small bistratified RGCs and associated interneurons that combine excitation from S-cones and inhibition from (L + M)-cones [39]. Therefore, the responses of ipRGCs activated by S-cones might be reduced by inhibition from (L + M)-cones on simultaneous exposure to blue and green light, and NIF functions might show subadditivity to some types of polychromatic light [32–34] and two simultaneous exposures to monochromatic light [29], as in the present study.

This study confirms for the first time that the subadditive response affects pupillary constriction during exposure to extremely short pulses of blue and green light. This response might be involved in the activation of cones, which provide input to the ipRGCs.

## Conclusion

We examined pupillary constriction during separate and simultaneous exposure to extremely short pulses of blue and green light of three irradiance intensities. We found that higher irradiance resulted in more pronounced pupillary constriction, with pupil constriction during exposure to a pulse of blue light being significantly greater than during exposure to a pulse of green light in all irradiance conditions. Interestingly, pupillary constriction during the simultaneous exposure to pulses of blue and green light was smaller than during exposure to a pulse of blue light despite the double irradiance intensity of the combination. This indicates that the effect of blue light on ipRGCs may be inhibited by simultaneous exposure to green light and shows the subadditive response in terms of pupillary constriction.

## Abbreviations

NIF: Non-image-forming
ipRGC: Intrinsically photosensitive retinal ganglion cell
LED: Light-emitting diode
KSS: Kwansei Gakuin Sleepiness Scale
VAS: Visual analog scale
PD: Pupil diameter
ANOVA: Analysis of variance
