# Correction to: Effect of simultaneous exposure to extremely short pulses of blue and green light on human pupillary constriction

**DOI:** 10.1186/s40101-018-0180-z

**Published:** 2018-08-30

**Authors:** Soomin Lee, Shougo Ishibashi, Yoshihiro Shimomura, Tetsuo Katsuura

**Affiliations:** 1 Center for Environment, Health and Field Sciences, Chiba University, 6-2-1, Kashiwanoha, Kashiwa, 277-0882 Japan; 2 Graduate School of Engineering, Chiba University, Chiba, Japan; 3 Present address: East Japan Railway Company, Tokyo, Japan

## Correction

Following publication of the original article [1], the authors reported that the abstract was missing from this article. The abstract text is included here in this Correction article in full:

**Abstract**

**Background:** Simultaneous exposure to blue and green light at night has been found to result in less melatonin suppression than monochromatic exposure to blue or green light. This effect is called the subadditive response to light. We conducted an experiment on the pupil response to extremely short pulses of blue and green light to examine whether the subadditive response affects pupillary constriction.

**Methods:** Eleven healthy young male volunteers participated in this study. Each subject sat in a chair with his head facing a diffusion panel located in front of an integrating sphere. Light-emitting diodes were arrayed in the integrating sphere. After 45 min of dark adaptation, the subject was exposed to a 1 ms pulse of blue (peak wavelength 470 nm) and/or green (532 nm) light with irradiance intensities of 10, 15, and 20 μW/cm^2^ simultaneously or separately. We measured pupil diameter and collected subjective evaluations during each of the nine lighting conditions.

**Results:** Higher irradiance resulted in more pronounced pupillary constriction. Pupil constriction during exposure to a pulse of blue light was significantly more pronounced than during exposure to a pulse of green light under all irradiance conditions. Pupillary constriction in response to simultaneous exposure to a pulse of blue light and a pulse of green light was less pronounced than during exposure to a pulse of blue light alone despite the double irradiance intensity of the combination.

**Conclusions:** This study confirms for the first time that the subadditive response had an effect on pupillary constriction during simultaneous exposure to extremely short pulses of blue light and green light.

**Trial registration:** Retrospectively registered.
